# Enhanced Recovery After Surgery Pathways and Obstetric Anesthesia: A Bibliometric Analysis

**DOI:** 10.7759/cureus.79038

**Published:** 2025-02-15

**Authors:** Abhijit Nair, Ujjwalraj I Dudhedia

**Affiliations:** 1 Anesthesiology, Ibra Hospital, Ibra, OMN; 2 Anesthesiology, Dr. L. H. Hiranandani Hospital, Mumbai, IND

**Keywords:** anesthesia, bibliometric analyis, enhanced recovery after surgery (eras) protocols, obstetric anesthesia, vos-viewer

## Abstract

As enhanced recovery after surgery (ERAS) pathways are being used exceedingly all over the world, research on ERAS and obstetric anesthesia is expanding. The necessity for uniform guidelines is highlighted by the notable regional and institutional differences in ERAS pathway implementation. Bibliometric research can identify these differences, which promotes a more consistent use of evidence-based procedures. The present bibliographic analysis reviewed 866 documents from the Scopus database using the keywords "enhanced recovery after surgery, ERAS, and Obstetrics Anesthesia." An increased number of articles were added to the database from 2017, with 175 articles in 2024. VOSviewer software (version 1.6.20, Leiden University, Netherlands) was used to investigate the various aspects of bibliometric analysis. The five aspects that were analyzed were co-authorship, co-occurrence, citation, bibliographic coupling, and co-citation. The United States of America had the maximum number of articles, citations, organizations, co-authorship, and co-citation with other authors, organizations, and countries. In the citations category, Gustafsson had the maximum number of citations in documents, and Anesthesia and Analgesia had the maximum number of citations in a journal. A thorough summary of the development of the field of ERAS in obstetric anesthesia can be found in this bibliometric analysis. This analysis has identified important research contributions, significant authors, and new trends by looking at publications, citations, and collaborations. Future research, policymaking, and clinical practice could benefit greatly from this information.

## Introduction and background

The multidisciplinary, evidence-based enhanced recovery after surgery (ERAS) perioperative care pathway aims to improve patient recovery after major surgeries. ERAS does not aim to replace traditional techniques, but to improve them. By re-evaluating traditional surgical approaches, ERAS integrates evidence-based modifications to reduce surgical stress, decrease complications, and promote early mobilization and discharge [1]. Preoperative counseling, nutrition optimization, reduced fasting period, multimodal analgesia, and early mobilization are among the fundamental tenets of ERAS. The ability of ERAS pathways to incorporate the best available evidence into clinical practice and guarantee a patient-centered approach to care is a cornerstone of their success [2].

Cesarean deliveries, which are among the most common surgeries performed globally, have seen an increasing implementation of ERAS protocols [3-5]. For the parturient safety and comfort, obstetric anesthesia, including general and regional anesthesia (such as spinal or epidural anesthesia), is important [6-8]. As both maternal and neonatal outcomes must be taken into account, cesarean deliveries pose unique challenges. In obstetric anesthesia, ERAS protocols place a high priority on factors like efficient pain management with minimal opioid use, early maternal mobility and breastfeeding initiation, and neonatal bonding. These protocols comprehend that mothers and newborns require customized care that addresses their physiological and psychological needs [9,10].

By examining patterns, trends, and networks within a particular field of study, bibliometric analysis is a type of quantitative study used to assess academic literature. To evaluate research productivity, citation dynamics, and collaborations between researchers, institutions, and nations, this approach utilizes statistical tools. Bibliometric studies highlight research gaps, identify influencing works, and offer insightful information about the evolution of knowledge, all of which help to direct future studies [11-14]. When it comes to ERAS in obstetric anesthesia, bibliometric analysis could show how research advances have occurred, how ERAS protocols have been adopted around the world, and how these protocols affect clinical outcomes.

This study aimed to analyze systematically the trends and hotspots related to ERAS practices in obstetric anesthesia across the world using relevant and validated tools.

## Review

Search strategy

We searched the literature from its inception to December 2024. Literature search and data downloads were done on a single day (28th December 2024) to minimize bias arising from database updates. We conducted a comprehensive search on the Scopus database (Elsevier Co., Amsterdam, Netherlands) using the following search strategy: (Title) OR (abstract) OR (keyword) = Enhanced Recovery After Surgery AND ERAS AND Cesarean Section AND Anesthesia. Articles that were included were clinical trials, systematic reviews and meta-analyses, case reports/series, clinical communications, and editorials. The Scopus file was stored in Comma Separated Values (csv) format. From the Scopus search, additional details like the number of publications per year, per author, per country, and organization were retrieved.

The information was exported for further analysis and included complete records and cited references. VOSviewer (version 1.6.20, Leiden University, Netherlands) was utilized for bibliometric analysis [15-17]. Using a VOSviewer, we performed five types of analysis. These analyses were co-authorship, co-occurrence, citation, bibliographic coupling, and co-citation. For co-authorship analysis, the units of analysis were authors, organizations, and countries [18]. For co-occurrence, the units of analysis were all keywords, author keywords, and index keywords [19]. For citation and bibliographic coupling, the units of analysis were documents, sources, authors, organizations, and countries [20,21]. For co-citation, the units of analysis were cited references, cited sources, and cited authors [22-54].

Results

Number of Publications Per Year Over the Years

**Figure 1:**
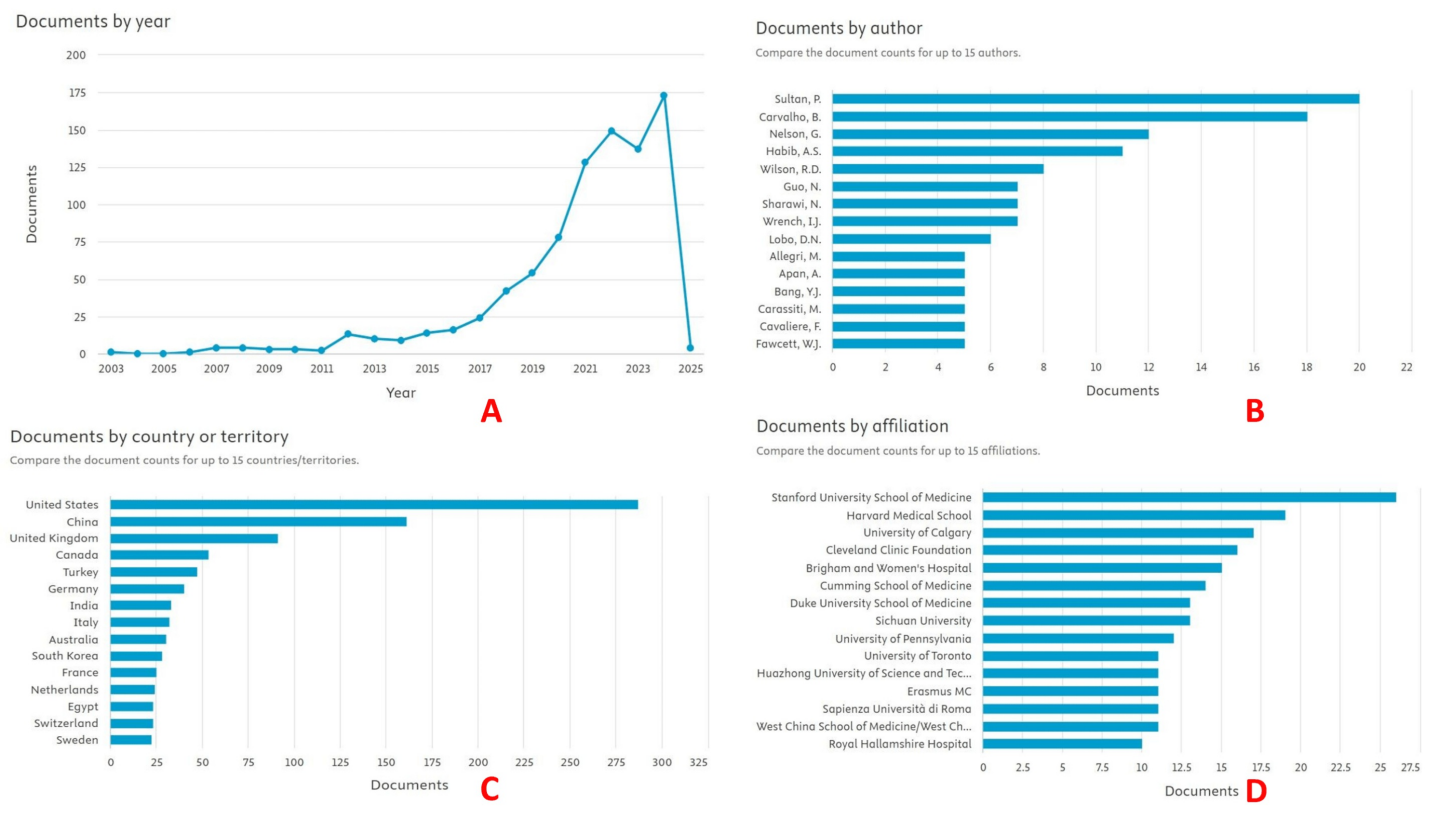
Scopus database search summary. (A) Documents by year, (B) Documents by author, (C) Documents by country or territory, and (D) Documents by affiliation. The image was generated using the Scopus database (Elsevier Co., Amsterdam, Netherlands) search (https://www.scopus.com/), using keywords: "Enhanced Recovery After Surgery AND ERAS AND Cesarean Section AND Anesthesia."

The Scopus search strategy generated results from the year 2003. A total of 866 documents were generated using the search strategy. However, from 2003 to 2017, the number of publications was consistently less than 25 per year. After 2017, there was a surge in the number of published articles, reaching a maximum of 175 in the year 2024 (Figure 1A). Sultan had the maximum number of documents on Scopus search (20), followed by Carvalho (18) and Nelson (12) (Figure 1B). Among countries, the United States of America (USA) had the maximum number of documents (287), and Stanford University School of Medicine had 26 documents among affiliations (Figures 1C, 1D).

Bibliometric Analysis Using VOSviewer

**Figure 2:**
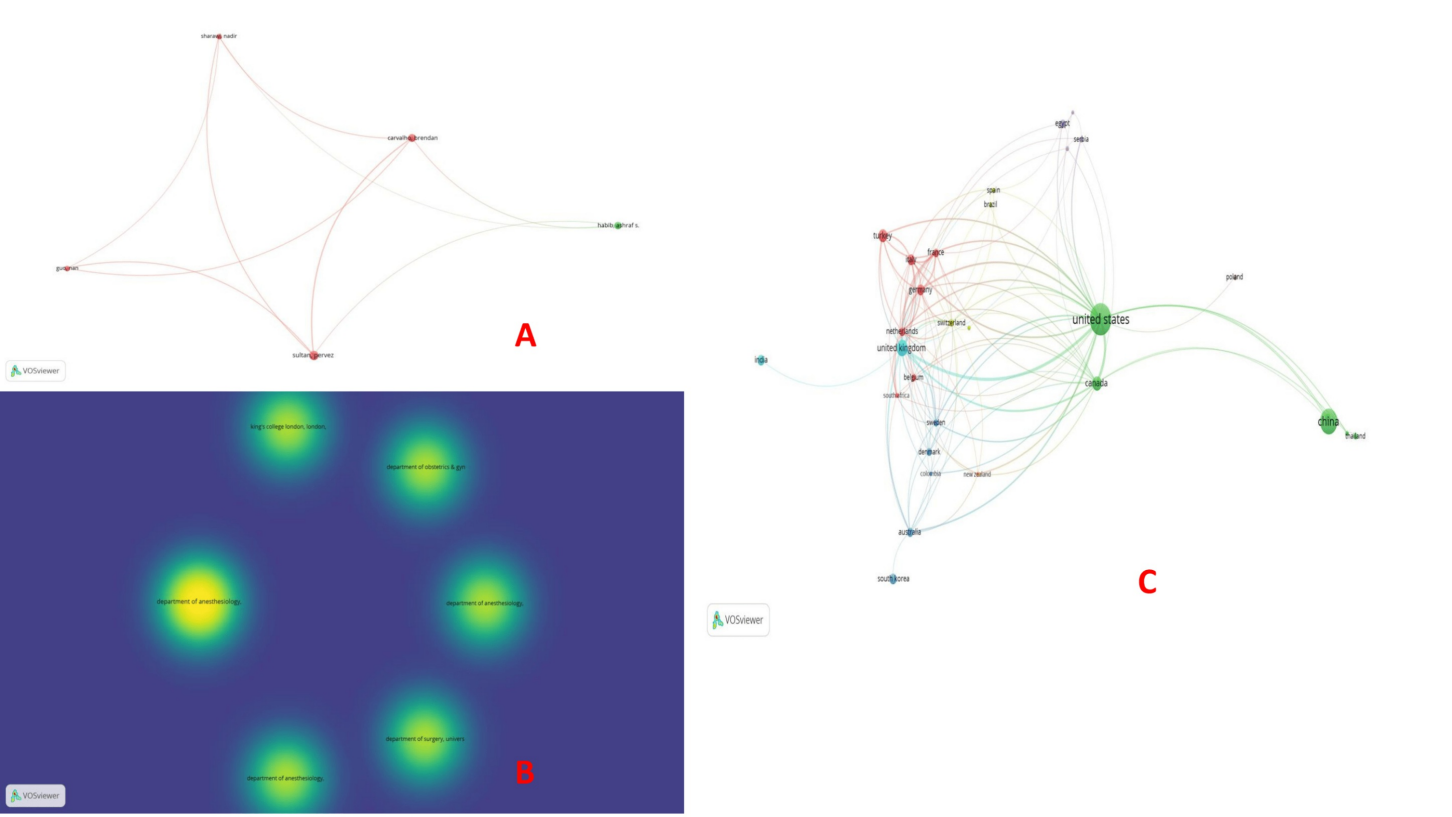
Co-authorship analysis. (A) Co-authorship and authors, (B) Co-authorship and organizations, and (C) Co-authorship and countries. The image was generated using the VOSviewer software (version 1.6.20, Leiden University, Netherlands) (VOSviewer-Visualizing scientific landscapes).

Co-authorship: VOSviewer identified 10 authors connected for co-authorship and authors. However, the largest set of connected items connected five of these authors (Sharawi, Carvalho, Habib, Sultan, and Guo) (Table 1, Figure 2A). Department of Anesthesiology, Perioperative and Pain Medicine, Brigham and Women’s Hospital, Harvard Medical School, Boston, United States had the maximum number of citations (1351), followed by the Department of Surgery, University of Virginia Health System, Charlottesville, USA (710) and Department of Obstetrics and Gynecology, Cumming School of Medicine, University of Calgary, Alberta, Canada (597), respectively (Figure 2B). In the co-authorship and countries category, the USA had the maximum co-authorship (250), followed by China (156) and the United Kingdom (UK) (67) (Figure 2C).

**Table 1:** Co-authorship and authors.

Author	Documents	Citations
Nelson [1,6]	10	739
Wilson [6]	6	627
Sultan [9]	14	374
Sultan [9]	5	118
Habib [9]	9	941
Carvalho [29]	6	168
Carvalho [29]	11	433
Sharawi [39]	5	152
Guo [40]	5	87
Metcalfe [41]	5	600

**Figure 3:**
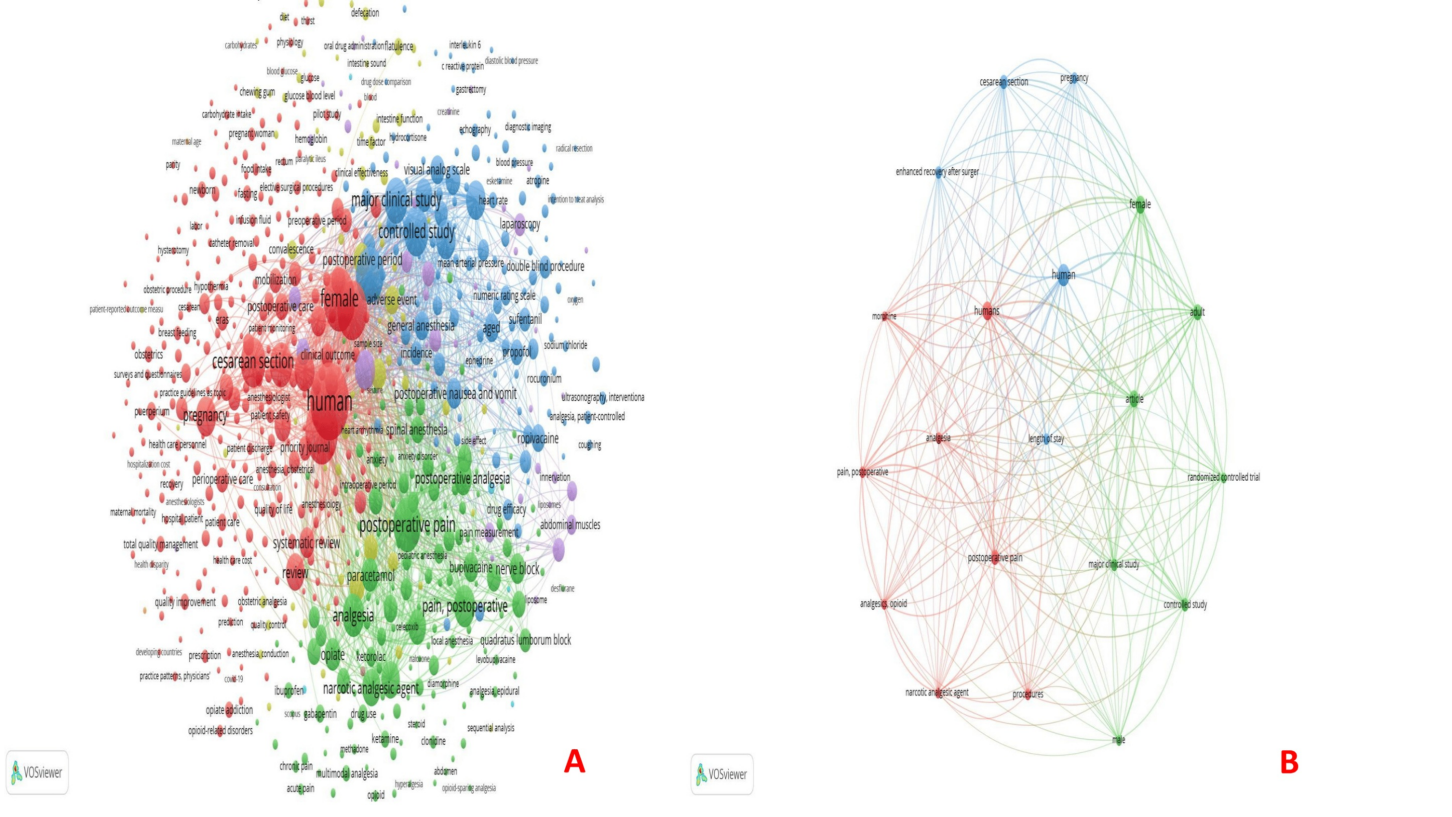
Keyword co-occurrence analysis. (A) Keyword co-occurrence and all keywords and (B) Keyword co-occurrence and 20 top keywords. The image was generated using the VOSviewer software (version 1.6.20, Leiden University, Netherlands) (VOSviewer-Visualizing scientific landscapes).

**Figure 4:**
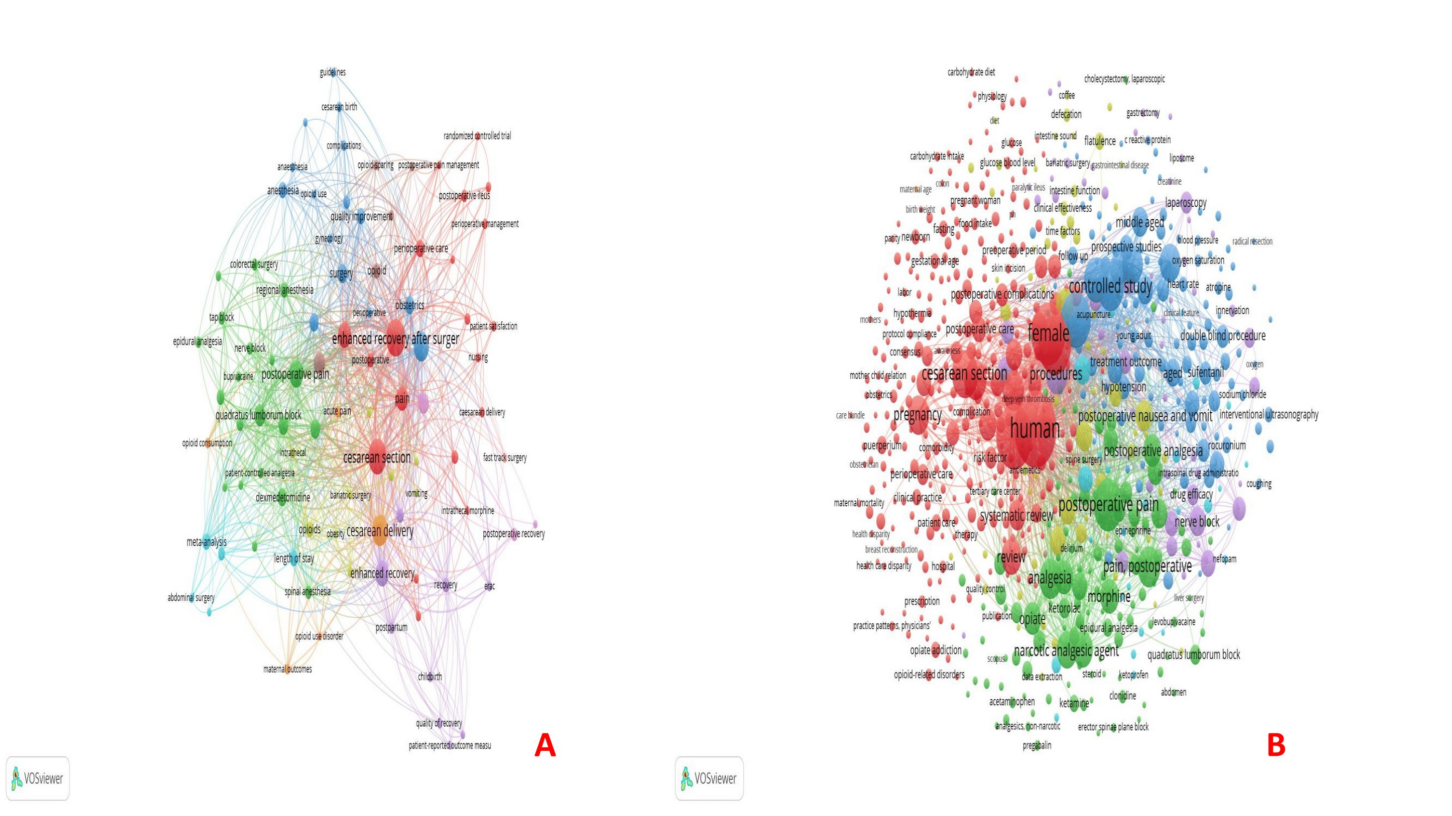
Keyword co-occurrence analysis (continued). (A) Keyword co-occurrence and author keywords and (B) Keyword co-occurrence and index keywords. The image was generated using the VOSviewer software (version 1.6.20, Leiden University, Netherlands) (VOSviewer-Visualizing scientific landscapes).

Keyword co-occurrence: It involves importing and preprocessing data, generating a co-occurrence matrix, visualizing the network, and interpreting the results. Of the 5158 keywords, 774 met the threshold. We initially created a network visualization using 774 keywords (Figure 3A). Subsequently, we selected the top 20 burst keywords (Table 2) and created another network visualization (Figure 3B). Among the author keywords and co-occurrences, "enhanced recovery after surgery" was the most common occurrence (88), followed by cesarean section (80) and next was cesarean delivery (58) (Figure 4A). Since index keywords serve as the basis for finding co-occurrences and building keyword networks, they are essential to bibliometric analysis. The precise words that define a document's content are known as index keywords. To make it easier to search for and retrieve pertinent literature, authors, indexers, or databases frequently assign these keywords. "Human" was the most occurring index keyword (634), followed by "humans" (454) and "female" (420) (Figure 4B).

**Table 2:** Co-occurrences and author keywords (top 20).

Keyword	Occurrences	Total link strength
Analgesia	41	56
Caesarean section	24	21
Cesarean delivery	58	88
Cesarean section	80	88
Enhanced recovery	41	64
Enhanced recovery after surgery	88	102
Eras	55	66
Length of stay	17	31
Meta-analysis	18	22
Multimodal analgesia	28	45
Obstetrics	21	33
Opioid	15	22
Opioids	15	28
Pain	36	45
Pain management	22	26
Perioperative care	20	24
Postoperative analgesia	23	28
Postoperative pain	49	62
Quadratus lumborum block	31	39
Transversus abdominis plane block	30	48

**Figure 5:**
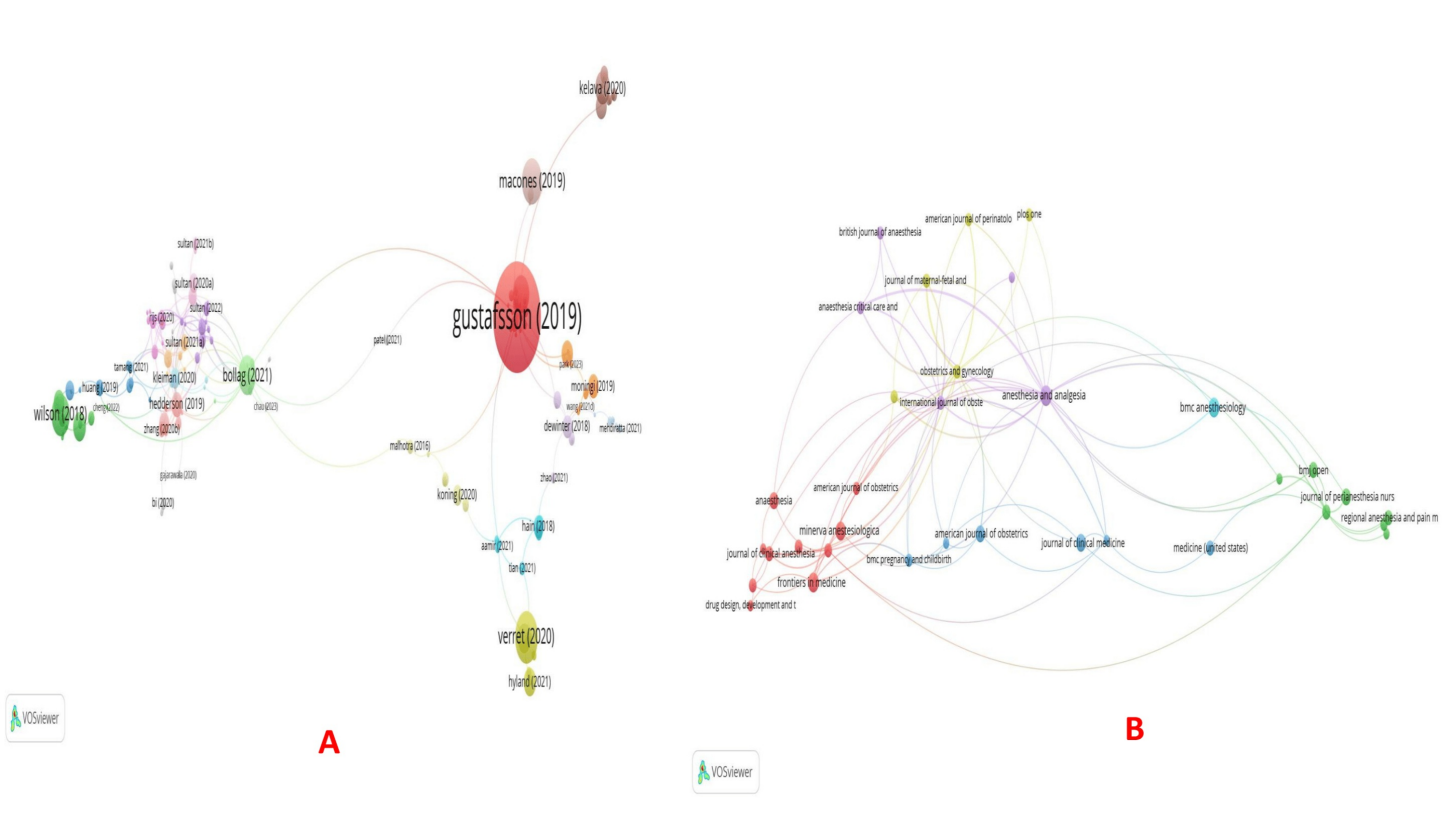
Citation analysis. (A) Citations and documents and (B) Citations and sources. The image was generated using the VOSviewer software (version 1.6.20, Leiden University, Netherlands) (VOSviewer-Visualizing scientific landscapes).

**Figure 6:**
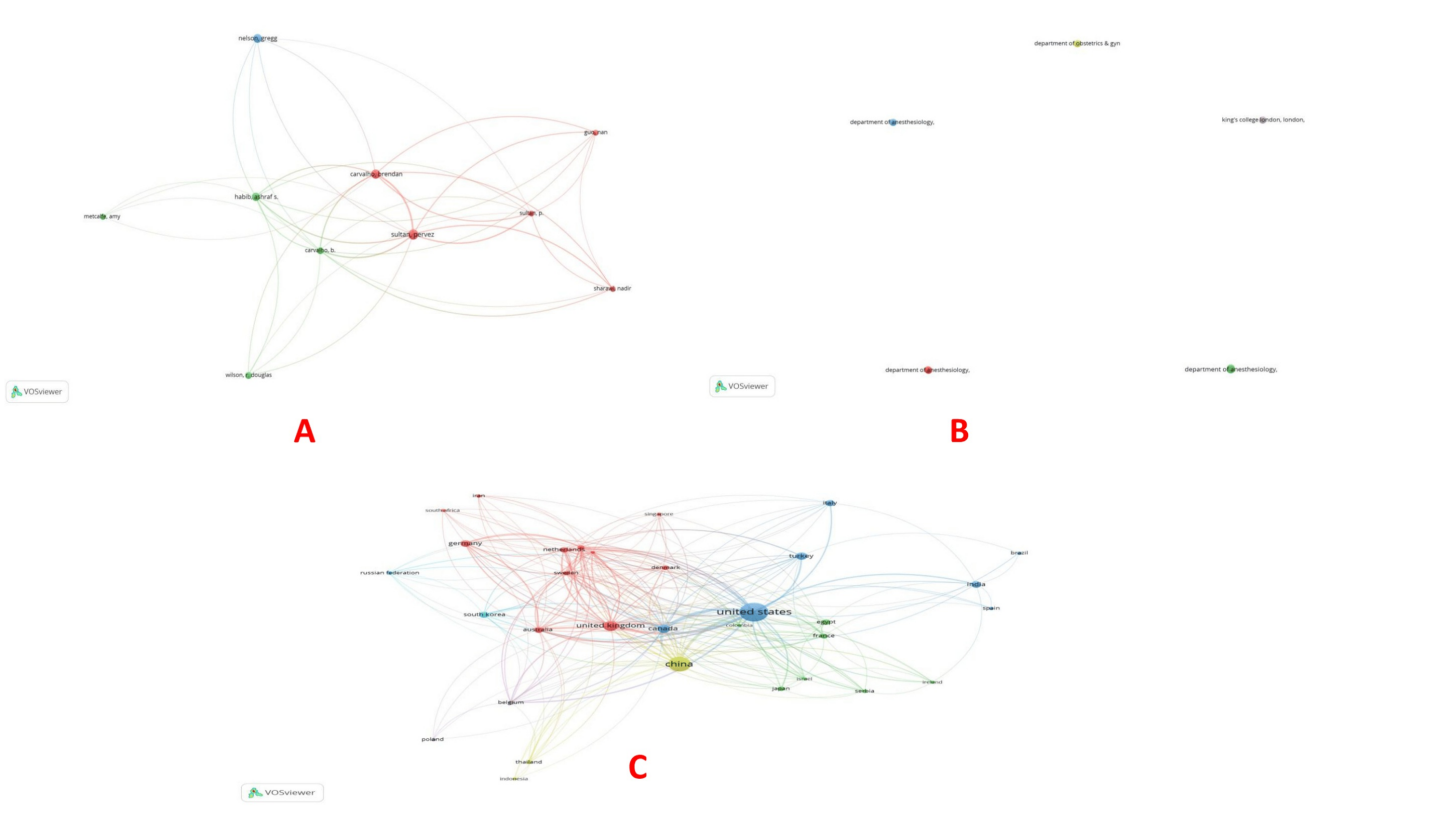
Citation analysis (continued). (A) Citations and authors, (B) Citations and organizations, and (C) Citations and countries. The image was generated using the VOSviewer software (version 1.6.20, Leiden University, Netherlands) (VOSviewer-Visualizing scientific landscapes).

Citations: As is obvious from the biggest node, the paper by Gustafsson (2019) received the maximum citations (1303), followed by the one by Verret (296) (Table 3, Figure 5A). The journal Anesthesia and Analgesia had 16 documents and 1132 citations. Also, the World Journal of Surgery had eight documents and 1412 citations (Figure 5B). Sultan had the maximum number of documents (14), followed by Carvalho and Nelson (11 and 10, respectively). However, the maximum number of citations received was for Habib (941), followed by Nelson (739) and Wilson (627) (Figure 6A). Among the top five organizations cited the most, four were from the USA, and one from the UK (Figure 6B).

**Table 3:** Citations and documents.

Document	Citations
Sultan (2020) [9]	38
Bollag (2021) [9]	168
Sultan (2021) [9]	33
Kleiman (2020) [28]	50
Ciechanowicz (2023) [42]	8
Ciechanowicz (2023) [42]	8
Gustafsson (2019) [43]	1303
Baluku (2020) [44]	24
Hedderson (2019) [45]	70
Verret (2020) [46]	296

Five organizations cited each other. They were: (i) Department of Anesthesiology, Duke University Medical Center, Durham, USA; (ii) Department of Anesthesiology, Perioperative, and Pain Medicine, Stanford University School of Medicine, Stanford, USA; (iii) Department of Surgery, University of Virginia Health System, Charlottesville, USA (maximum citations of 710); (iv) King's College London, United Kingdom; and (v) Stanford University School of Medicine, Stanford, USA. In terms of the number of documents, the USA has the maximum number (250), followed by China (156) and the UK (67). The maximum citations were also for documents from the USA (8563), followed by the UK (2988) and Australia (1798) (Figure 6C).

**Figure 7:**
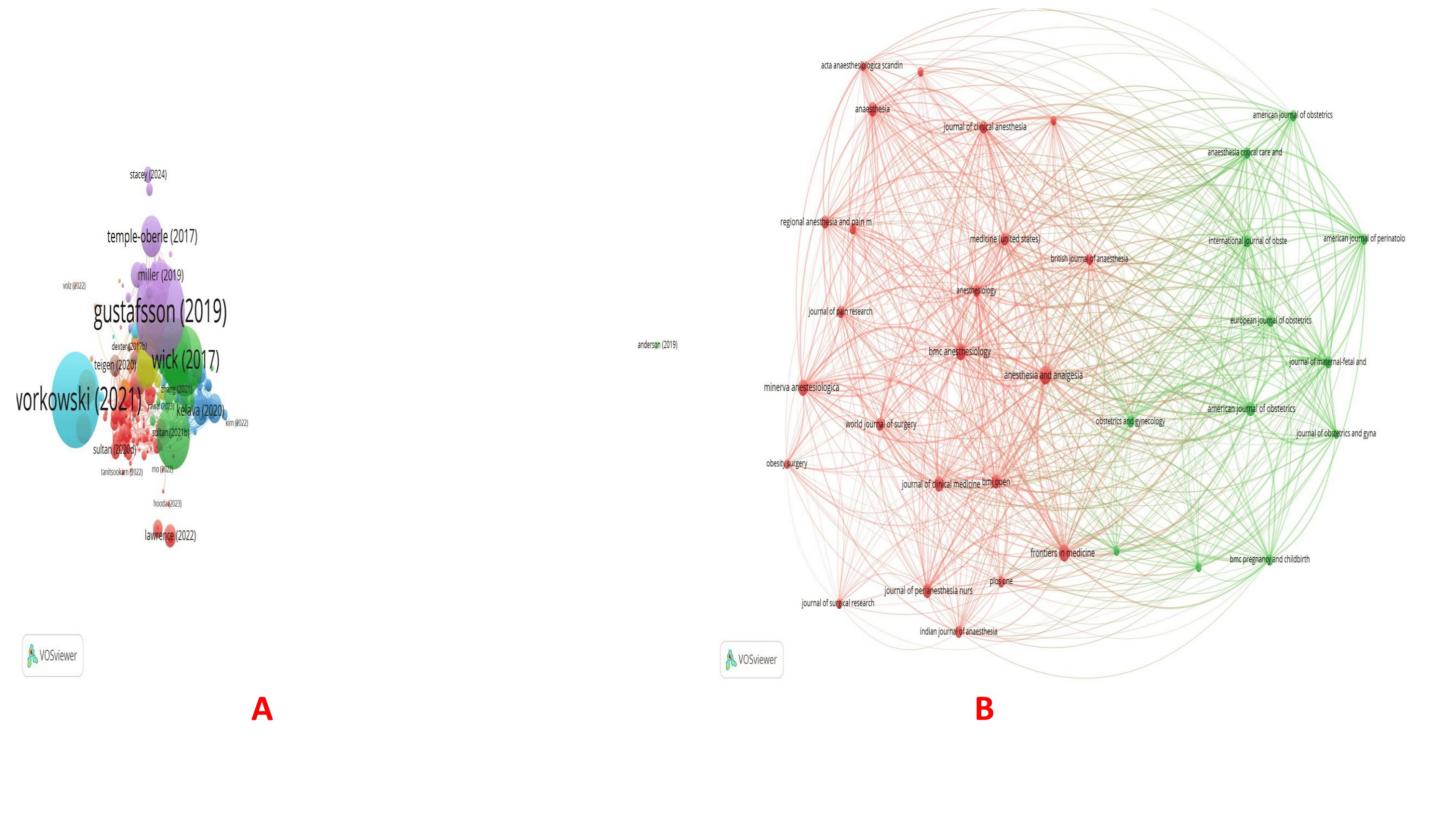
Bibliographic coupling analysis. (A) Bibliographic coupling and documents and (B) Bibliographic coupling and sources. The image was generated using the VOSviewer software (version 1.6.20, Leiden University, Netherlands) (VOSviewer-Visualizing scientific landscapes).

**Figure 8:**
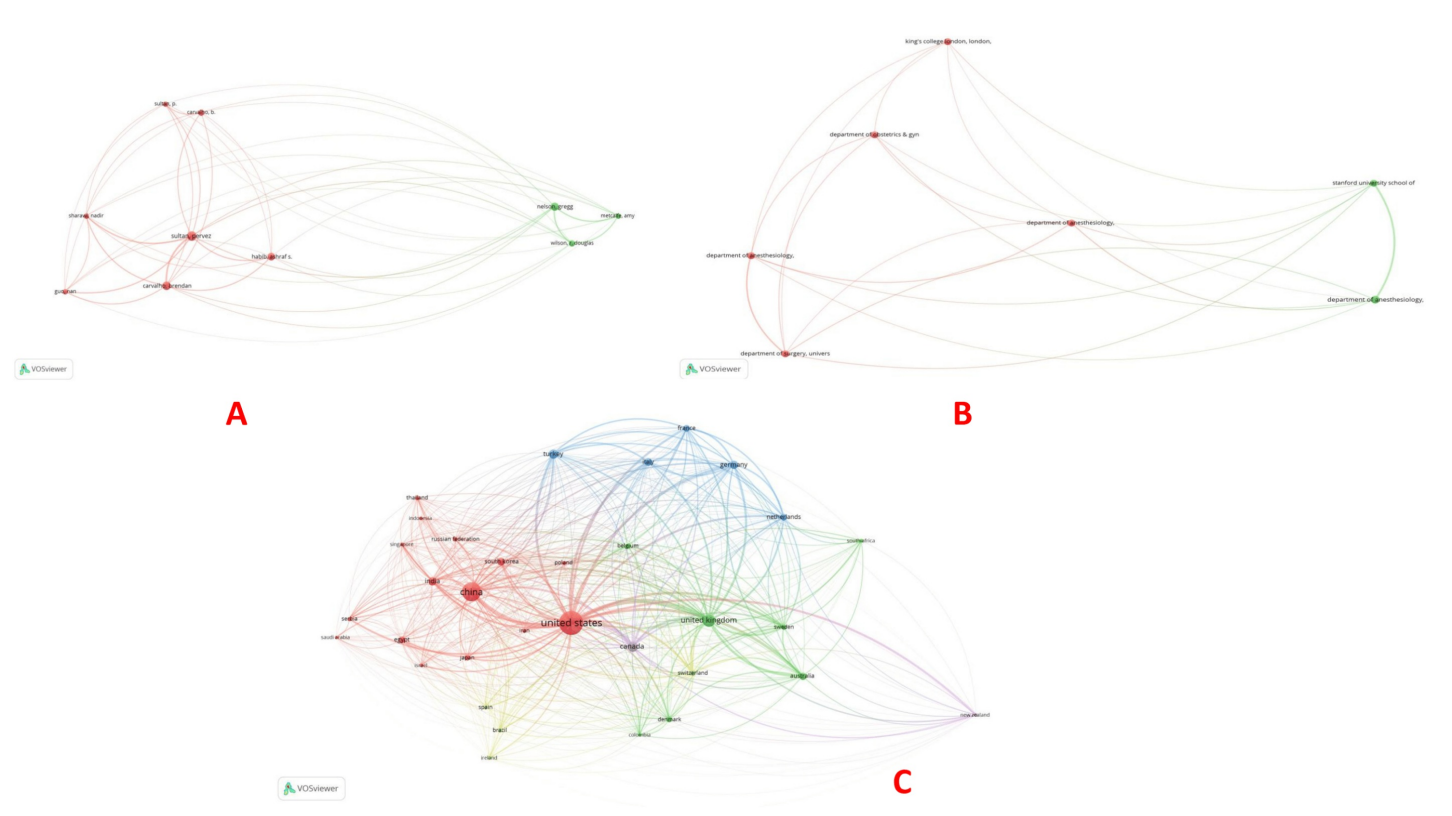
Bibliographic coupling analysis (continued). (A) Bibliographic coupling and authors, (B) Bibliographic coupling and organizations, and (C) Bibliographic coupling and countries. The image was generated using the VOSviewer software (version 1.6.20, Leiden University, Netherlands) (VOSviewer-Visualizing scientific landscapes).

Bibliographic coupling: Bibliographic coupling is a method of network analysis used in bibliometrics. Two documents are said to be bibliographically coupled if they share one or more references. Kessler first introduced this concept in 1963, and it has since become an essential tool in analyzing the structure of scientific literature. Bibliographic coupling is done for the documents, sources, authors, organizations, and countries. The documents by Bollag (2023) had 168 citations, followed by Peahl (2019), which had 44 citations (Table 4, Figure 7A). Among sources, Anesthesia and Analgesia had the maximum number of citations (1132), followed by the American Journal of Obstetrics and Gynecology (827) and Anesthesiology (627) (Figure 7B). The highest influence on bibliographic coupling with authors was by Sultan et al., Carvalho et al., and Nelson et al. based on the total link strength (Figure 8A). Stanford University School of Medicine had five documents but 70 citations for bibliographic coupling and organizations. On the contrary, with four documents, Harvard Medical School, Boston, had 1351 citations (Figure 8B). Bibliographic coupling was observed in a total of 33 countries. The USA had the biggest node, followed by China and the UK (Figure 8C).

**Table 4:** Bibliographic coupling and documents.

Document	Citations	Total link strength
Huang (2019) [6]	33	20
Bollag (2021) [9]	168	27
Ciechanowicz (2023) [42]	8	49
Peahl (2019) [47]	44	30
Sangkum (2021) [48]	13	34
Meng (2021) [49]	24	45
Holland (2020) [50]	3	35
Irani (2023a) [51]	21	146
Irani (2023b) [51]	24	145
O'carroll (2022) [52]	11	39

**Figure 9:**
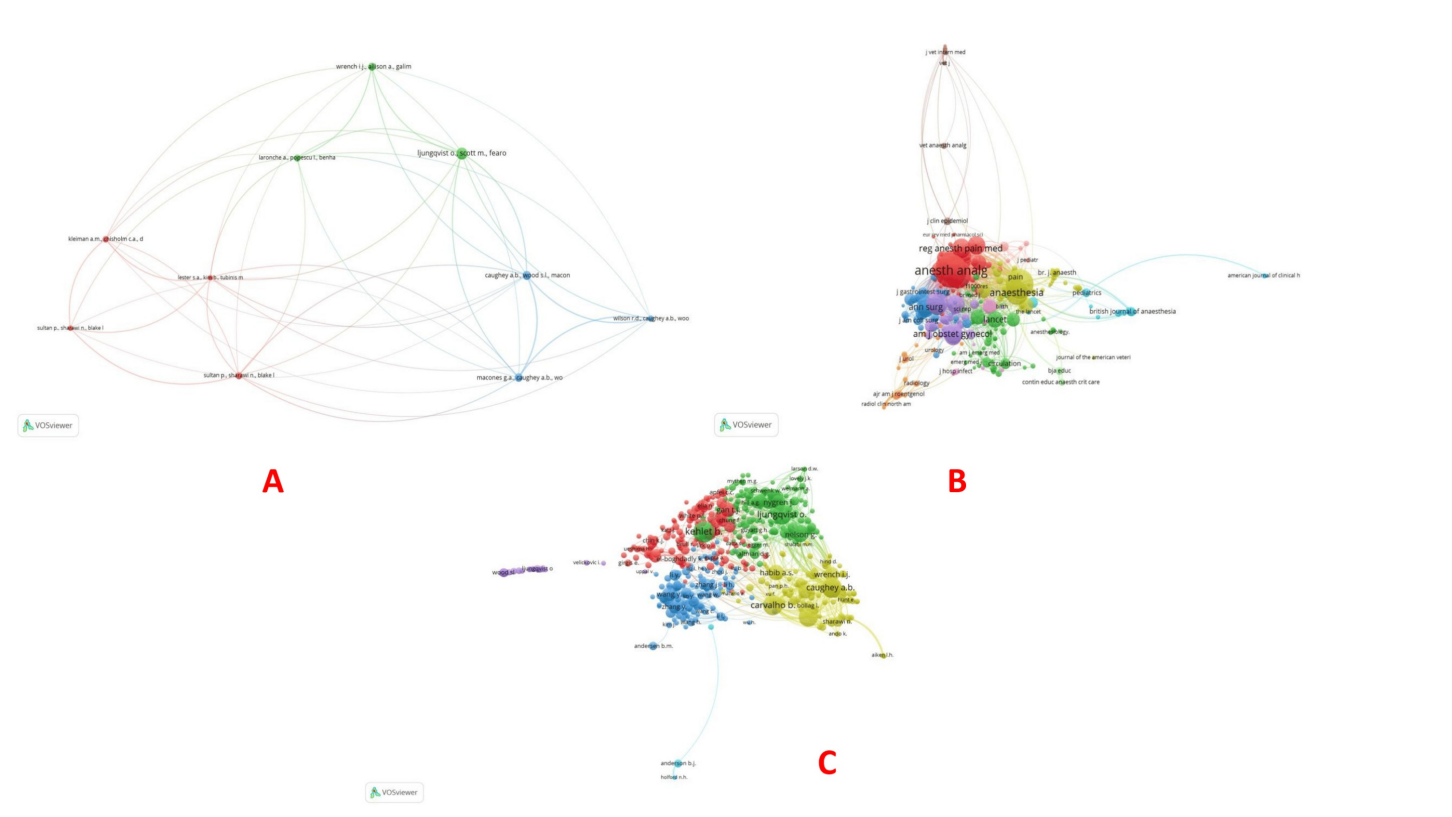
Co-citation analysis. (A) Co-citation and cited references, (B) Co-citation and cited sources, and (C) Co-citation and cited authors. The image was generated using the VOSviewer software (version 1.6.20, Leiden University, Netherlands) (VOSviewer-Visualizing scientific landscapes).

Co-citation: Ljungqvist et al. (2017) had the maximum co-citations (33) [2], followed by the seminal article by Kehlet published in 1997, which had 21 co-citations [8] (Table 5, Figure 9A). Anesthesia and Analgesia had the maximum co-citations (1427), followed by anesthesiology (1091) and the British Journal of Anesthesia (BJA) (1025) (Figure 9B). Carvalho had the maximum co-citations (346), followed by Ljungqvist (338) and Caughey (323) (Figure 9C).

**Table 5:** Co-citation and cited references.

Cited reference	Citations	Total link strength
Ljungqvist et al. (2017) [2]	33	31
Wilson et al. (2018) [4]	10	32
Caughey et al. (2018) [5]	20	50
Kehlet (1997) [8]	21	14
Bollag et al. (2021) [9]	12	19
Sultan et al. (2020) [39]	12	19
Macones et al. (2019) [41]	17	41
Macones et al. (2019) [41]	14	13
Laronche et al. (2017) [53]	12	22
Wrench et al. (2015) [54]	15	23

Discussion

Primary Findings

In this study, we used the VOSviewer software to perform bibliometric analysis on the trends and hotspots research related to ERAS and obstetric anesthesia using the Scopus database. This bibliometric analysis analyzed 866 publications retrieved from the database search. To our knowledge, this is the first-ever bibliometric analysis performed to access research dynamics related to ERAS and obstetric anesthesia. We noticed that the number of publications increased after the year 2021. The maximum publications were from the USA. Stanford University School of Medicine had the maximum number of publications. Sultan and Carvalho were the most influential authors. In bibliographic coupling, documents by Bollag had the maximum citations, and the journal Anesthesia Analgesia had the maximum citations. As an author, Sultan had the maximum number of citations, Standard University School of Medicine had the maximum number of citations from organizations, and the USA had the maximum number of citations among countries. The paper by Ljungqvist had maximum co-citations from cited sources. Anesthesia Analgesia had the maximum number of citations, and Carvalho had the maximum co-citations among authors.

ERAS is a protocolized scientific method to lessen the surgical stress response by altering inflammatory and metabolic alterations. It comprises multimodal evidence-based approaches at every stage of perioperative care [23]. The use of ERAS pathways in obstetric surgeries is an important development. Cesarean sections are now carried out using ERAS protocols, commonly referred to as enhanced recovery after cesarean sections (ERAC) [9,24]. Previously, these surgeries used conventional methods that frequently involved extended fasting and delayed mobilization. ERAS pathways prioritize multimodal analgesia, early mobilization, and a quicker recovery of gastrointestinal function. Patients can achieve functional recovery faster by reducing recovery-delaying factors like prolonged fasting and excessive opioid use. This results in shorter hospital stays following cesarean delivery, facilitating mothers to return home and commence caring for their newborns sooner quickly [25-28].

Significant cost savings tend to be a result of shorter hospital stays, less medication use, and a decreased need for admission and interventions. Additionally, ERAS procedures lessen the possibility of postoperative complications, which have been linked to higher medical costs. This is particularly significant in obstetric populations, where families and healthcare systems could encounter less financial strain due to expedited, evidence-based care pathways [29-32].

Bibliometric analysis is a quantitative research technique used to assess academic literature. It does so by examining patterns, trends, and networks within a particular field of study by assessing research productivity, citation dynamics, and collaborations between researchers, institutions, and countries using validated tolls. Bibliometric studies highlight research gaps, identify influential works, and offer insightful information about the advancement of knowledge, all of which help to direct future studies [33-38]. When it comes to ERAS in obstetric anesthesia, bibliometric analysis can show how research has progressed, how ERAS protocols have been adopted around the world, and how these protocols affect clinical results.

A comprehensive overview of the development of the field can be found in this bibliometric analysis of ERAS in obstetric anesthesia. This analysis has identified noteworthy research contributions, significant authors, and new trends by looking at publications, citations, and collaborations. Future studies, policymaking, and clinical practice can all benefit significantly from this information. The findings of the present study suggest that ERAS research in obstetric anesthesia is getting significant attention, which is consistent with the implementation of the pathways. The necessity for consistent guidelines has been emphasized by the substantial regional and institutional differences in ERAS pathway implementation. Bibliometric research can identify these differences, which promotes a more consistent use of evidence-based practices.

Given the fact that ERAS procedures have shown significant advantages, there are certain difficulties in incorporating them into obstetric anesthesia. Potential obstacles include the necessity for interdisciplinary collaboration opposition to modifying long-standing practices, and variations in institutional resources. Furthermore, a careful balancing of treatment priorities is required because of the simultaneous focus on maternal and newborn health. Future research should focus on improving ERAS protocols to ensure that they are applicable and effective in a variety of healthcare settings. In addition, by tracking improvements in maternal and neonatal care outcomes, bibliometric analysis can measure the influence of such advancements.

This bibliometric analysis has several limitations. We searched only the Scopus database. The other databases that could also be used for bibliometric analysis are the Web of Science and PubMed. Therefore, it is quite possible that some articles featuring our search strategy could have been missed in the analysis. In addition, the keywords used for the search were only in English. Therefore, publications in which keywords or abstracts were not in English were excluded from the study. We analyzed our data by downloading the Scopus CSV file on 28th December 2024. As many articles get added to the database periodically, it is quite possible that we could not include those articles in the analysis that got included in the database after the above-mentioned date.

## Conclusions

The implementation of ERAS pathways with patient-centered care is an added advantage in obstetric anesthesia. Improved maternal satisfaction and an easier perioperative experience can be achieved by incorporating shared decision-making, preoperative education, and emotional support. This bibliometric analysis has attempted to highlight the landscape of ERAS in obstetric anesthesia by identifying research trends, influential publications, and collaboration networks. By facilitating a deeper understanding of impactful practices, bibliometric studies can contribute to refining ERAS protocols, promoting global standardization, and ultimately improving maternal and neonatal outcomes.
